# 1α,25-Dihydroxyvitamin D3 accelerates skin wound re-epithelialization by promoting epidermal stem cell proliferation and differentiation through PI3K activation: an *in vitro* and *in vivo* study

**DOI:** 10.1590/1414-431X2025e14121

**Published:** 2025-03-03

**Authors:** Rongshuai Yan, Zhihui Liu, Song Wang, Dongli Fan

**Affiliations:** 1 Department of Plastic Surgery, Xinqiao Hospital, Third Military Medical University (Army Medical University), Chongqing, China; 2 State Key Laboratory of Trauma, Burn and Combined Injury, Institute of Burn Research, Southwest Hospital, Third Military Medical University (Army Medical University), Chongqing, China; 3 Department of Burn and Plastic Surgery, General Hospital of Central Theater Command, Wuhan, China

**Keywords:** 1α,25-Dihydroxyvitamin D3, Epidermal stem cell, Proliferation, Differentiation, Wound healing

## Abstract

1α,25-Dihydroxyvitamin D3 (VD3), the active form of vitamin D, plays a crucial role in wound healing. In this study, we aimed to investigate the effect of VD3 on the proliferation and differentiation of epidermal stem cells (EpSCs) and monitor its impact on re-epithelialization. We established a murine full-thickness skin defect model and applied four doses of VD3 (0, 5, 50, and 250 ng/mouse/day) to the wounds topically for three days. Immunostaining and flow cytometry confirmed the effect of VD3 on the proliferation and differentiation of EpSCs in wounds. This effect of VD3 (0, 1, 10, and 50 nM) on EpSCs and its possible mechanism were further confirmed *in vitro* by CCK8, westen blot, immunostaining, and flow cytometry. We found that on day five post-wounding, the means±SD length of the neo-epidermis was 195.88±11.57, 231.84±16.45, 385.80±17.50, and 268.00±8.22 μm in the control, 5, 50, and 250 ng groups, respectively, with a significant difference from the control (all P<0.05). Immunostaining and flow cytometry showed that VD3 improved the proliferation and differentiation of K15+ EpSC (*vs* control, all P<0.05), K14+ epidermal progenitor cells (*vs* control, all P<0.05), and K10+ epidermal terminal cells (*vs* control, all P<0.05) *in vivo* and *in vitro*. The PI3K signaling pathway appeared to underlie this response because significant inhibition of the response was found when inhibitors were used to inhibit PI3K. Our study demonstrated that VD3 is a potent promoter of cutaneous wound healing by stimulating EpSC proliferation and differentiation through PI3K activation.

## Introduction

Wound healing is a complex program with the cooperation of various cells, growth factors, and signals (1,2). Epidermal stem cells (EpSCs) reside in the basal layer of the interfollicular epidermis and the bulge region of hair follicles (HFs) and sebaceous glands (3,4). After skin injury, EpSCs are guided by various growth factors and signals to proliferate, differentiate, and migrate toward the center of the wound. Without the endogenous stimulation of EpSCs, the healing of wounds is delayed, while exogenous supplements to the wounds promote wound healing (5,6). These results showed that EpSCs are indispensable in maintaining epidermal homeostasis and responding to wounds, but the mechanism involved in wound repair is still not fully understood.

Accumulating pieces of evidence demonstrate that 1α,25-dihydroxyvitamin D3 (VD3), the active form of vitamin D, and its receptor provide significant signaling to promote wound healing (7). This signaling appears to be an essential mechanism to regulate epidermal proliferation, migration, and differentiation and to affect wound healing. *In vitro* studies have unveiled that supplements of VD3 and its analogs regulate the migration, proliferation, and gene expression of both fibroblasts and keratinocytes, two cells involved in healing cutaneous wounds (8,9). Numerous retrospective and prospective cohort hospital-based studies have indicated the beneficial effect of VD3 on wound healing (10). *In vivo* studies have demonstrated that applying VD3 topically on full-thickness cutaneous wounds and diabetic wounds (11,12) could affect wound healing considerably. However, few studies focused on the effect of VD3 on EpSCs and whether the effect contributed to the wound healing process.

Therefore, we investigated the responses of EpSCs to VD3. We established a murine full-thickness skin defect model and evaluated the topical use of VD3 on the wounds.

## Material and Methods

### Assay for skin wound healing

**Figure 1:**
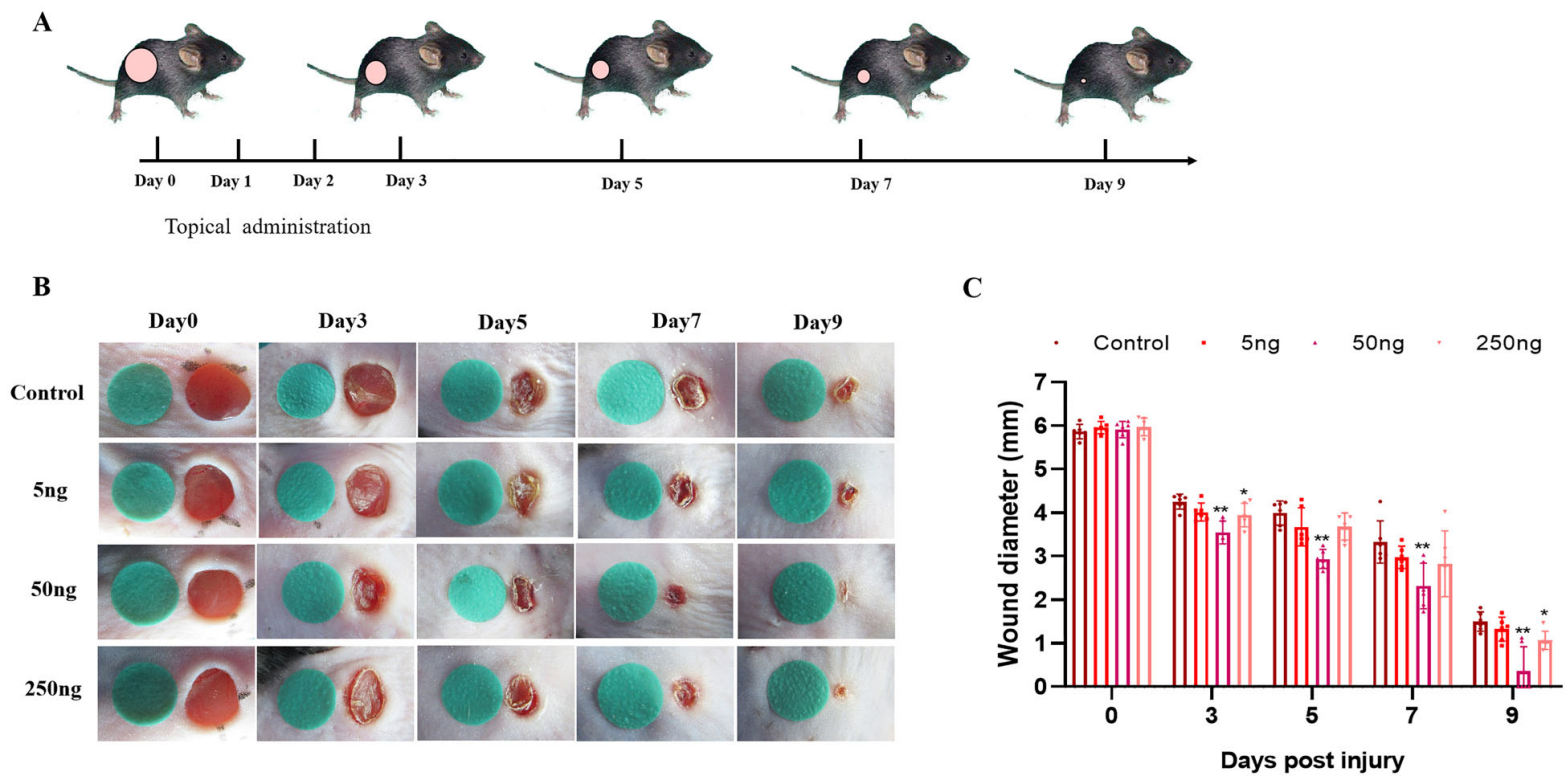
Vitamin D3 promoted skin wound healing in a dose-dependent manner. **A**, Schedule of wound treatment. **B**, Representative macroscopic images of wounds treated with different doses of vitamin D. The diameter of the green circle is 6 mm. **C**, Quantitative analysis of wound area (%) at each time point (n=6 mice/group). Data are reported as means±SD. *P<0.05 and **P<0.01 compared with control group (ANOVA).

The procedure was performed as previously described (13). Male C57BL/6J mice (4-6 weeks of age) were used. Mice were anesthetized by intraperitoneal injection of 1% pentobarbital (0.01 mg/g body weight). Then, after shaving the hair from the dorsal surface of the mouse and cleaning, two full-thickness excisional skin wounds were made using a 6-mm biopsy punch. The mice were randomly assigned to four groups, each with six animals. Each mouse received a daily topical application of 50 μL PBS with different doses of VD3 (Sigma, D1530, USA) immediately after surgery for 3 days (Figure 1A). We chose ten subcutaneous injection sites as shown in Supplementary Figure S1, and each site received 5 μL VD3 of different concentrations: i) control group (50 μL PBS); ii) 5 ng group (50 μL PBS with 5 ng VD3); iii) 50 ng group (50 μL PBS with 50 ng VD3); and iv) 250 ng group (50 μL PBS with 250 ng VD3). Four doses of VD3 (0, 5, 50, and 250 ng/mouse per day) were applied for 3 days (Figure 1A). Wounds were photographed with a digital camera on days 0, 3, 5, 7, and 9. The diameter of each wound was measured using ImageJ 1.48 software (NIH, USA) and the average was calculated for each animal.

### Isolation and culture of primary mouse EpSCs

Isolation of EpSCs from 3-day-old C57BL/6J mice was performed using standard protocols as described in previous studies (14). Briefly, the dorsal skin of the mice were incubated in 0.5% Dispase II solution (Roche; 04942078001, Switzerland) overnight at 4°C to separate the epidermis from the dermis. The epidermis was then dissociated into a single-cell suspension by gently pipetting up and down in 0.25% trypsin for 10 min. The trypsin was neutralized with calcium-free RPMI 1640 medium containing 10% FBS, and the cell suspension was then filtered through a 70-mm cell strainer to form a pellet. The cell pellet was then resuspended in a special EpSCs medium consisting of K-SFM (Gibco; 17005, USA) with 30 mg/mL bovine pituitary extract, 10 ng/mL mouse epidermal growth factor (BD, USA; 354001), 1×10^-10^ M cholera toxin (Sigma; C9903), 0.05 mM calcium chloride, and 100 IU/L penicillin and streptomycin solution (Gibco; 15140122).

Cells were then counted and seeded onto dishes precoated with type IV collagen (Sigma; C5533) for 10 min at 37°C. Non-adherent cells were discarded by gentle washing with warm PBS, and the culture medium was changed every 2-3 days. Cells at passage 3 were used for subsequent experiments.

### Hematoxylin-eosin (H&E) staining and analysis

The mice were sacrificed on the 5th, 7th, and 21st day after wounding to collect wound, kidney, and liver samples for analysis. The samples were sequentially fixed with 4% paraformaldehyde, embedded in paraffin, sectioned, mounted on slides, and stained with H&E. The stained sections were then photographed using a microscope (LEICA, Germany, CTR6000) and measured using ImageJ 1.48V software (NIH, USA).

### Immunohistochemistry and immunofluorescence staining

For H&E staining, wound specimens were processed as previously described (15). The unstained sections were further processed by sequential deparaffinization, rehydration, antigen retrieval with boiling citrate buffer (10 mM, pH 6.0), and blocking with 3% BSA (bovine serum albumin). Sections were then incubated with primary antibody (PCNA, 1:500, ab18197; Abcam, UK) overnight at 4°C. Signals were visualized using avidin-peroxidase reagent (SP-9001, Zhongshan Biology Company, China) in coordination with 3,30-diaminobenzidine tetrahydrochloride (DAB) chromogen (ZLI-9017, Zhongshan Biology Company). For immunofluorescence (IF) staining, cells were fixed with 4% paraformaldehyde (PFA) for 20 min at room temperature. The cells were then washed three times with PBS, blocked with 10% donkey serum, and incubated with primary antibodies (K15, Abcam, 1:200, ab52816; K14, Proteintech, China; 1:200, 60320-1-Ig; K10, Abcam, 1:200, ab76318) overnight at 4°C. Signals were visualized with secondary antibodies and photographed with a microscope (LEICA, CTR6000).

### Flow cytometry analysis

After the cells were isolated, the appropriate primary antibody was added and incubated at 4°C for 1 h. Data were analyzed using the Attune Acoustic FocUsing Cytometer (Applied Biosystems, Life Technologies, USA) in conjunction with FlowJo software (Tree Star Incorporation, USA). The antibodies used were CD49f-BV421 (1:200, BioLegend, 313624, USA), CD71-PE (1:200, Invitrogen, 2024832, USA), K15-PE (1:200, Santa Cruz Biotechnology, sc-47679, USA), K14-AF488 (1:200, Santa Cruz Biotechnology, sc-53253), and K10-PE (1:200, Santa Cruz Biotechnology, sc-53251). Samples were then fixed, permeabilized, and stained according to a standard protocol. Experiments were repeated at least three times under identical conditions and settings.

### Western blot analysis

On days 5 after wounding, wound samples were crushed in liquid nitrogen and homogenized using the whole cell lysis kit (Keygen, KGP2100, China). After centrifugation at 4^o^C at 12,000 *g* for 15 min, the supernatants were collected and measured using the BCA assay (Pierce, 23225, USA). Forty milligrams of each sample were then sequentially separated on a 10% SDS-PAGE gel and electrophoretically transferred to polyvinylidene difluoride (PVDF) membranes (Millipore, USA). The membranes were blocked with 3% BSA, incubated with primary antibodies (PI3 Kinase p85 Antibody, CST, 4292; Phospho-PI3 Kinase p85 (Tyr458)/p55 (Tyr199), CST, 17366) at 4°C overnight, and incubated with horseradish peroxidase-conjugated secondary antibodies at room temperature for one hour. Finally, signals were visualized and photographed using an enhanced chemiluminescence (ECL) detection kit (Pierce, 35055) and the Molecular Imager ChemiDoc TMXRS+Imaging System (BioRad, USA).

### Proliferation assay

The procedure was performed as previously described (16). Briefly, mouse EpSCs were seeded onto three replicates in 96-well plates in eSCs medium. After the cells were cultured for 48 h and incubated at 37°C with the addition of 10 μL/well of CCK8 reagent (CK04, Dojindo, Japan), their absorbance was measured at 450 nm using an enzyme-linked immunosorbent assay reader (UV-3600, Shimadzu, Japan).

### Statistical analysis

Data are reported as means±SD and analyzed by one-way analysis of variance (ANOVA), and the difference between groups was analyzed using LSD with SPSS 18.0 software. P<0.05 was considered statistically significant.

## Results

### VD3 accelerated cutaneous wound closure in a dose-dependent manner

Macroscopic examination of wounds showed that VD3 significantly enhanced wound closure compared to the vehicle control in a dose-dependent manner (Figure 1B). No significant difference was observed in the diameter of the wounds among all the groups immediately after the surgery. The means±SD diameter of wounds in the four groups was 5.86±0.17, 5.96±0.14, 5.91±0.19, and 5.97±0.20 mm. At the other time points, the 50-ng VD3 group displayed the smallest diameter of the wounds (day 3 diameter=3.54±0.26 mm, day 5 diameter=2.93±0.22 mm, day 7 diameter=2.31±0.53 mm, day 9 diameter=0.36±0.56 mm), and the 50-ng dose accelerated wound closure compared with the control group (P<0.01). On day 9 post-wounding, the means±SD diameter of wounds was 1.50±0.22, 1.32±0.28, 0.36±0.56, 1.06±0.21 mm in the control, 5, 50, and 250 ng groups, respectively. The 250-ng dose also accelerated wound closure compared to control on day 3 (P=0.033) and day 9 (P=0.043). However, the 5-ng dose did not reach statistical significance *versus* the control. These results demonstrated that VD3 promoted cutaneous wound healing in a dose-dependent manner.

**Figure 2:**
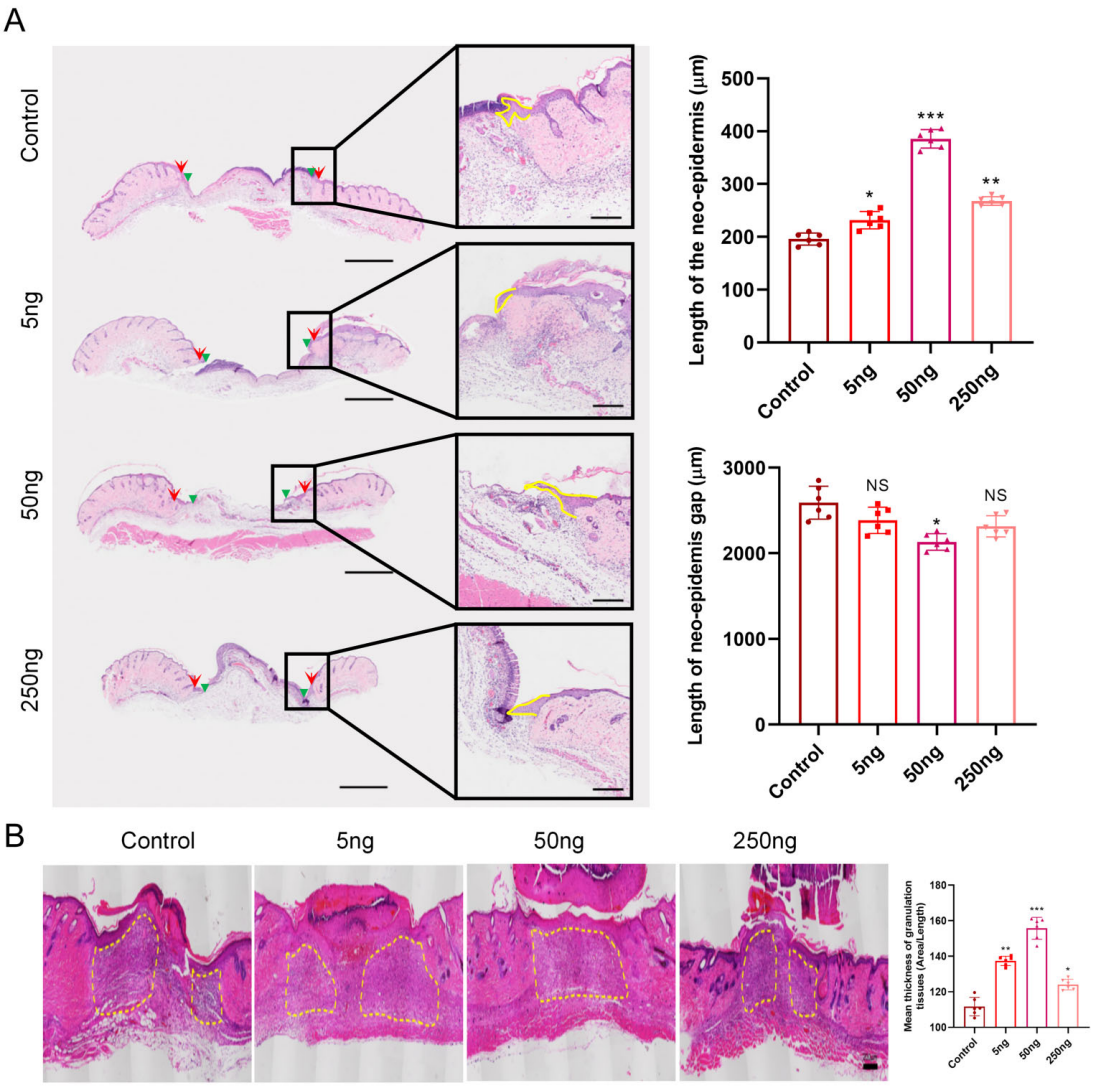
Vitamin D3 enhanced re-epithelialization and granulation tissue formation in a dose-dependent manner. **A**, Representative hematoxylin and eosin (H&E) images of re-epithelialization from each group 5 days after wounding. Scale bars: 1000 μm (low magnification); 200 μm (high magnification). Quantitative analysis of neo-epidermal length (n=6 mice/group) and neo-epidermal gap (n=6 mice/group). Red arrows indicate the wound edges; green arrowheads indicate the tips of the epithelial tongues; yellow lines outline the neo-epidermis. **B**, Representative H&E images of granulation tissue from each group on day 7 after wounding. Scale bar: 200 μm. Quantitative analysis of granulation tissue thickness (n=6 mice/group). Data are reported as means±SD. *P<0.05, **P<0.01, and ***P<0.001 compared with control group; NS, non-significant (ANOVA).

H&E staining revealed that VD3 significantly increased re-epithelialization, as shown by longer neo-epidermal length and smaller neo-epidermal gaps (Figure 2A) compared to control on day 5 post-wounding. The means±SD length of the neo-epidermis was 195.88±11.57, 231.84±16.45, 385.80±17.50, and 268.00±8.22 μm in the control, 5, 50, and 250 ng groups, respectively. All VD3 doses had a positive effect on the neo-epidermis, with a significant difference *versus* the control (all P=0.000). Regarding the length of the neo-epidermis gap, all other groups showed a significant difference compared with the control group (5 ng *vs* control P=0.024, 50 ng *vs* control P=0.000, 250 ng *vs* control P=0.004).

Analysis of granulation tissue thickness in H&E-stained sections showed that VD3 significantly augmented granulation tissue formation by day 7 post-injury across all doses *versus* the control (all P=0.000) (Figure 2B).

To assess the potential toxicity *in vivo* of the four doses of VD3, we observed the animal behavior for 21 days. At 21 days post-surgery, we obtained the liver and kidney for histological analysis. During the 21 days, no mouse died, and we did not observe any change in animal behavior with the four doses of vitamin D. As shown in Supplementary Figure S2, histological analysis revealed no significant abnormalities or damage of the liver and kidney, indicating negligible toxicity *in vivo* of the four doses of vitamin D.

Taken together, these results demonstrated that VD3 expedited wound closure by enhancing two key processes - re-epithelialization and granulation tissue formation - in a dose-dependent manner.

### VD3 promoted proliferation and differentiation of EpSCs in wounds

**Figure 3:**
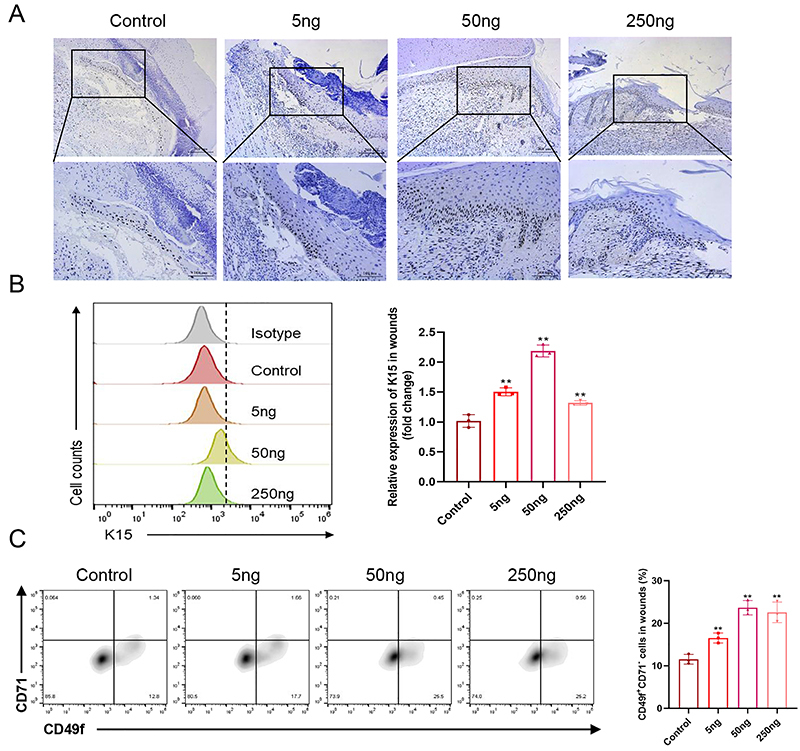
Vitamin D3 promoted proliferation and differentiation of epidermal stem cells (EpSCs) in wounds. **A**, Representative immunohistochemical-stained sections of PCNA expression. Scale bars: 200 μm (low magnification); 100 μm (high magnification). K15+ (**B**) and CD49f+/CD71- (**C**) cells from wound tissues on the 5th day after wounding treated with different doses of vitamin D (0, 5, 50, 250 ng) were detected by flow cytometry (n=3/group). Data are reported as means±SD. **P<0.01 compared with control group (ANOVA).

To elucidate how VD3 enhances the re-epithelialization stage of wound healing, we examined its effects on the proliferation and differentiation of EpSCs in wounds. Immunohistochemical staining showed markedly increased numbers of PCNA+ proliferating epidermal cells in VD3-treated wounds compared to control wounds on day 5 post-injury (Figure 3A). Flow cytometry confirmed dose-dependent increases in the expression of K15 in wounds with a significant difference *versus* the control (5 ng *vs* control P=0.000, 50 ng *vs* control P=0.000, 250 ng *vs* control P=0.002) (Figure 3B). Additionally, compared to wounds treated with the control vehicle, VD3-treated wounds had a significantly increased ratio of CD49+CD71- cells in a dose-dependent manner (5 ng *vs* control P=0.007, 50 ng *vs* control P=0.000, 250 ng *vs* control P=0.000) (Figure 3C).

**Figure 4:**
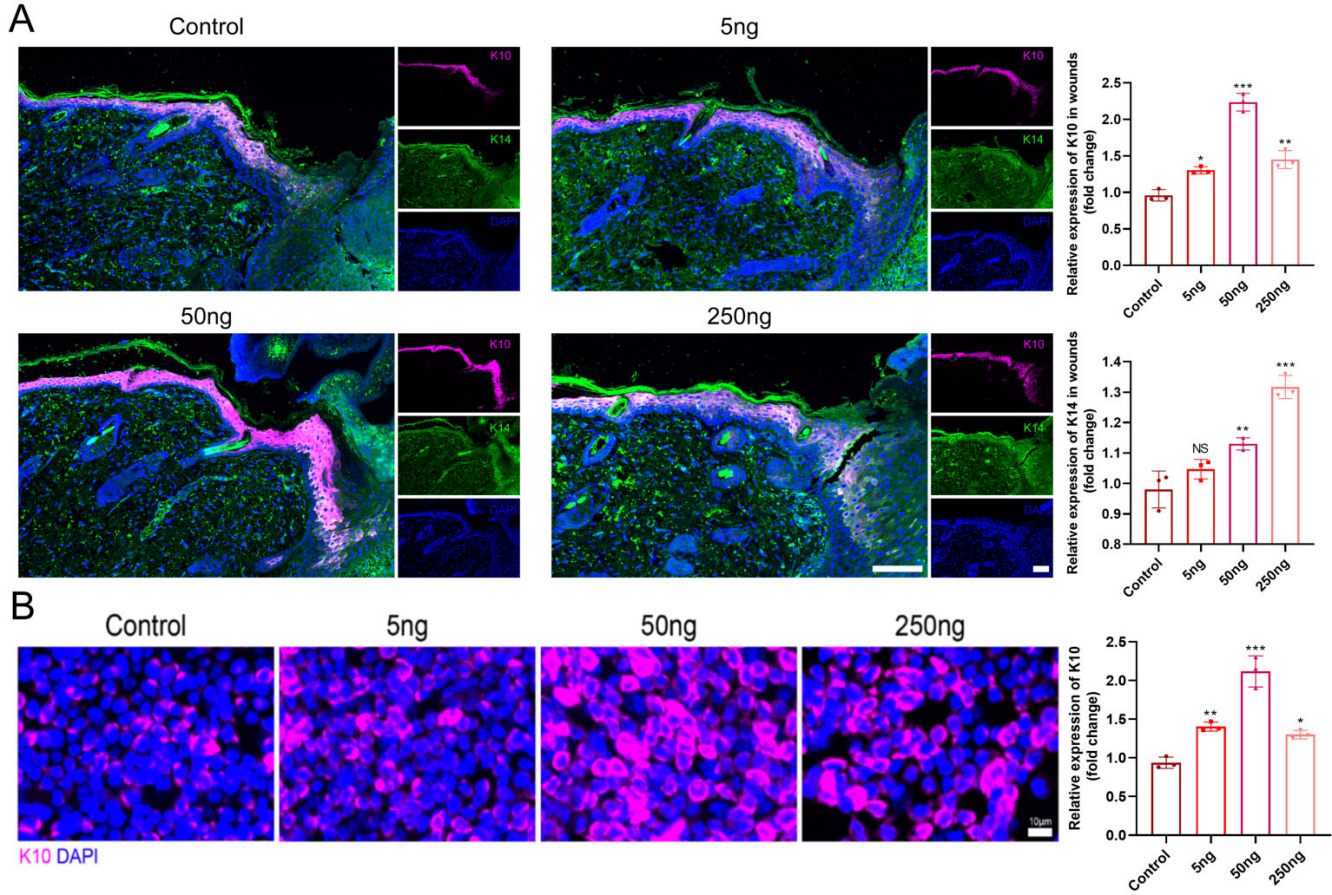
Vitamin D3 promoted the differentiation of epidermal stem cells (EpSCs) in wounds. Representative immunofluorescence-stained sections for co-expression of (**A**) K10 and K14 in wound tissues (scale bars: 100 μm) and the expression of (**B**) K10 in wound epidermis (scale bar: 10 μm) on the 5th day after wounding, and quantitative data (right panels) (n=3/group). Data are reported as means±SD. *P<0.05, **P<0.01, and ***P<0.001 compared with control group; NS: non-significant (ANOVA).

Co-immunostaining revealed increased expression of both K10 (a marker of terminally differentiated cells) (5 ng *vs* control P=0.003, 50 ng *vs* control P=0.000, 250 ng *vs* control P=0.000) and K14 (a marker of progenitor cell) (5 ng *vs* control P=0.079, 50 ng *vs* control P=0.002, 250 ng *vs* control P=0.000) in VD3-treated wounds compared to control wounds on day 5 (Figure 4A). Consistently, the immunofluorescence results from the wounded epidermis also confirmed that VD3 upregulated the expression of K10 in a dose-dependent manner (5 ng *vs* control P=0.001, 50 ng *vs* control P=0.000, 250 ng *vs* control P=0.005) (Figure 4B). These data provided compelling evidence that VD3 acted by inducing EpSCs differentiation to generate keratinocytes that drive re-epithelialization during cutaneous wound repair.

These results indicated that VD3 stimulated both the proliferation and differentiation of EpSCs in a dose-dependent manner *in vivo*.

### VD3 promoted proliferation and differentiation of EpSCs *in vitro*

**Figure 5:**
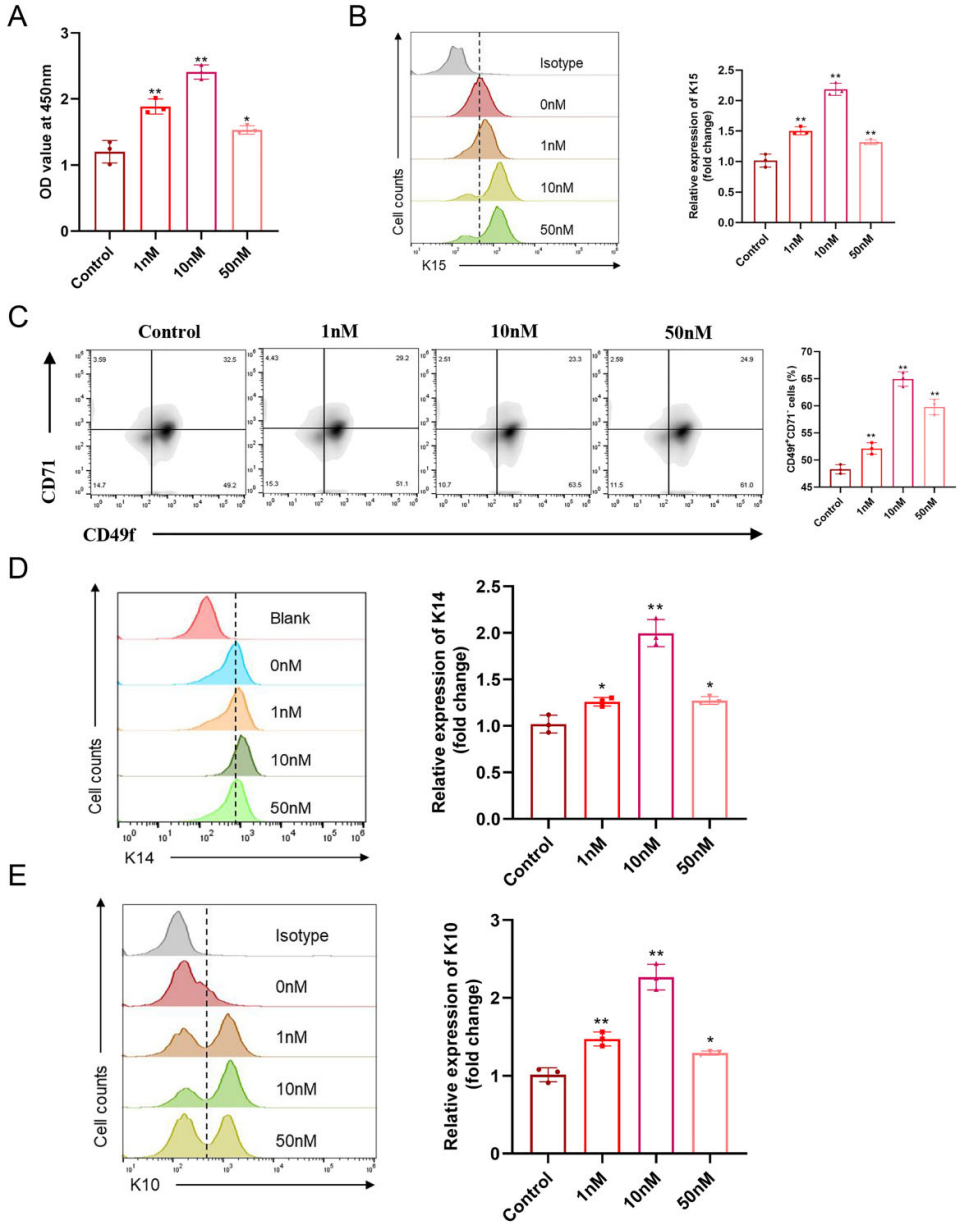
Vitamin D3 promoted proliferation and differentiation of epidermal stem cells (EpSCs) *in vitro*. **A**, The proliferation of skin epidermal stem cells from C57BL/6 mice treated with different doses of vitamin D3 (0, 1, 10, 50 nM) for 48 h was detected by CCK8 (n=3/group). K15+ (**B**), CD49f +/CD71- (**C**), K14+ (**D**), and K10+ (**E**) cells were detected by flow cytometry after the EpSCs were treated with different doses of vitamin D (0, 1, 10, 50 nM) for 48 h, and quantitative data (right panel) (n=3/group). Data are reported as means±SD. *P<0.05 and **P<0.01 compared with control group (ANOVA).

The *in vitro* CCK8 assay (Figure 5A) further confirmed that VD3 dose-dependently increased proliferation in EpSCs (1 nM *vs* control P=0.000, 10 nM *vs* control P=0.000, 50 nM *vs* control P=0.010). Flow cytometry confirmed dose-dependent increases in the expression of K15 *in vitro* (1 nM *vs* control P=0.000, 10 nM *vs* control P=0.000, 50 nM *vs* control P=0.002) (Figure 5B). Additionally, compared to control groups treated with 0 nM VD3, VD3-treated wounds had a significantly increased ratio of CD49+CD71- cells in a dose-dependent manner (1 nM *vs* control P=0.004, 10 nM *vs* control P=0.000, 50 nM *vs* control P=0.000) (Figure 5C). Furthermore, flow cytometry results also showed VD3 dose-dependently increased the expression level of K14 (1 nM *vs* control P=0.013, 10 nM *vs* control P=0.000, 50 nM *vs* control P=0.010) and K10 (1 nM *vs* control P=0.001, 10 nM *vs* control P=0.012, 50 nM *vs* control P=0.000) in isolated primary EpSCs cultures *in vitro* (Figure 5D and E). Collectively, VD3 had a large contribution in the proliferation and differentiation of EpSCs.

### PI3K was involved in VD3-induced proliferation and differentiation of EpSCs

**Figure 6:**
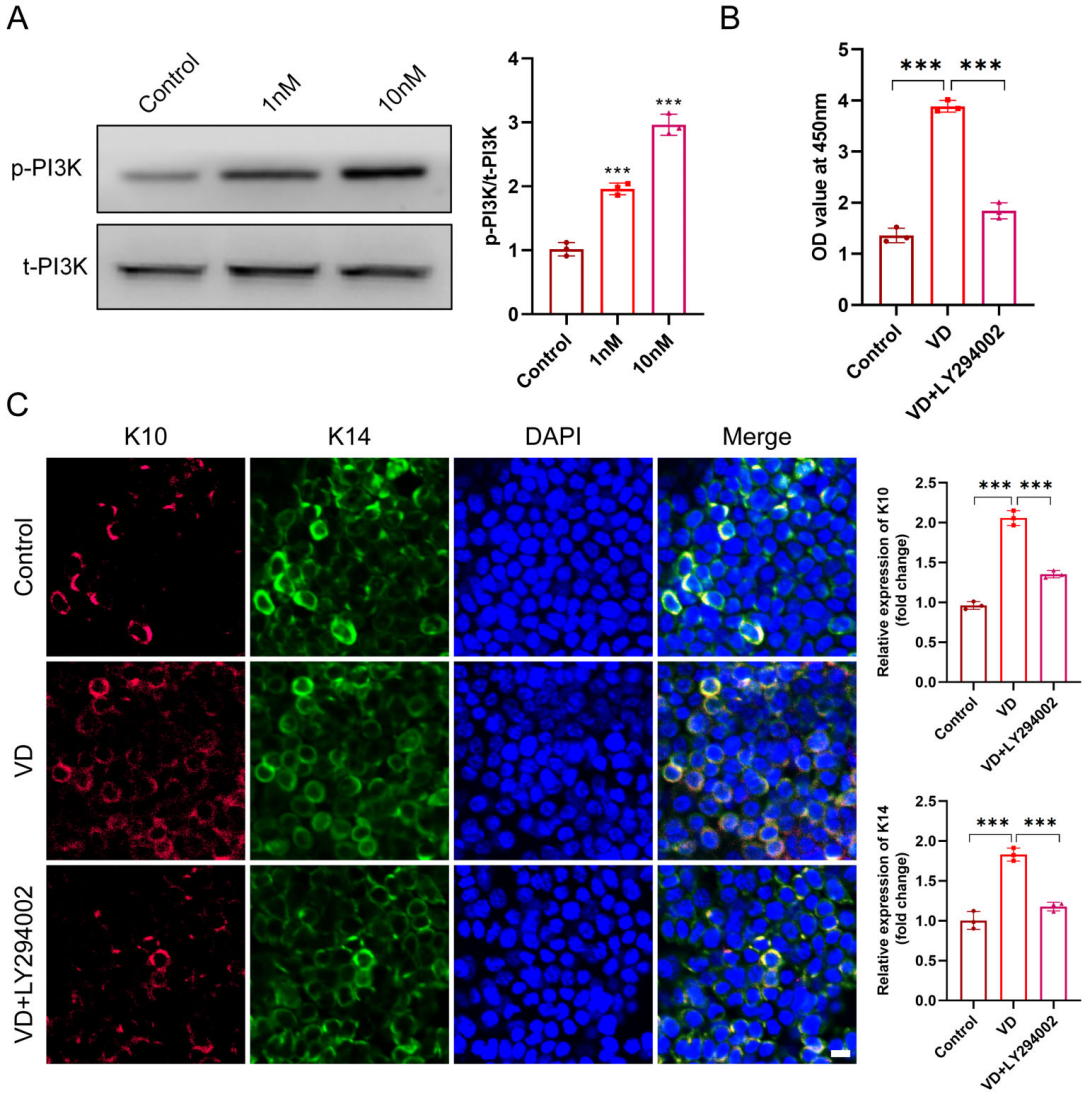
Vitamin D enhanced the proliferation and differentiation of epidermal stem cells by activating PI3K signaling pathway. **A**, Representative immunoblotting for the expression of p-PI3K and t-PI3K (n=3/group). **B**, The proliferation of skin epidermal stem cells treated with vitamin D or LY294002 for 48 h was detected by CCK8 (n=3/group). **C**, Representative immunofluorescence-stained sections (scale bars: 10 μm) for co-expression of K10 and K14 in wound epidermis tissues at day five after wounding, and quantitative data (right panel) (n=3/group). Data are reported as means±SD. ***P<0.001 compared with control groups (ANOVA).

To investigate the mechanism underlying VD3-mediated proliferation and differentiation of epidermal stem cells, we analyzed the PI3K signaling pathway associated with cell proliferation and differentiation (17). Immunoblotting results showed that VD3 increased the ratio of p-PI3K to t-PI3K compared to the untreated group (10 nM *vs* control P=0.000, 10 nM *vs* LY294002 P=0.000), suggesting that VD3 could activate the PI3K signaling pathway in a dose-dependent manner (Figure 6A). We found that VD3-induced proliferation of epidermal stem cell was significantly blocked when the cells were incubated with VD3 and LY294002 (10 nM *vs* control P=0.000, 10 nM *vs* LY294002 P=0.000) (Figure 6B), suggesting VD3 could facilitate the proliferation of epidermal stem cell via activating PI3K signaling pathway. Furthermore, immunofluorescence results showed that VD3 significantly upregulated the expression of K10 (10 nM *vs* control P=0.000) and K14 (10 nM *vs* control P=0.000) in wounds. Additionally, this was weakened by LY294002 (K10 LY294002 *vs* 10 nM P=0.000, K14 LY294002 *vs* 10 nM P=0.000) (Figure 6C), implying that the PI3K signaling pathway might contribute to the differentiation of epidermal stem cells. Our data indicated that VD3 enhanced the proliferation and differentiation of epidermal stem cells via activating the PI3K signaling pathway *in vivo* and *in vitro*.

## Discussion

EpSCs, which can produce differentiated cells and propagate to maintain a constant pool of EpSCs by dividing symmetrically or asymmetrically in the skin, play a fundamental role in wound healing (18). Accumulating evidence indicates that VD3 can regulate epidermal cell proliferation (8,9), migration (19), and differentiation (20) and affect wound healing (21).

In this study, we demonstrated that vitamin D acted through a novel mechanism to promote cutaneous wound healing by regulating EpSC proliferation and differentiation. Using a murine full-thickness excisional wound model, we showed that topical vitamin D significantly enhanced wound closure by stimulating re-epithelialization and granulation tissue formation. Notably, we revealed that vitamin D stimulated the proliferation and differentiation of EpSCs into epidermal keratinocytes, essential for re-epithelialization. Our findings provide compelling evidence that vitamin D promoted cutaneous regeneration upon injury by modulating EpSCs behavior.

Re-epithelialization is a critical stage of wound healing, requiring coordinated proliferation and migration of keratinocytes across the wound bed. Rapid re-epithelialization limits infection risk and fluid loss while also providing signals for downstream healing events (22). Our results showed that vitamin D strongly induced keratinocyte proliferation *in vivo*, as evidenced by increased PCNA expression (Figure 3A). This corroborates previous findings that vitamin D stimulates keratinocyte growth (8). Importantly, our study newly revealed that vitamin D elicited these effects on keratinocytes by regulating EpSCs, the stem cell population that sustains epidermal renewal. Specifically, vitamin D expanded the pools of EpSC markers like CD49f+/CD71- and K15+ cells while upregulating K14+ transient amplifying progenitors and differentiated K10+ keratinocytes. The ability of vitamin D to stimulate EpSCs proliferation was further verified in isolated primary EpSCs *in vitro*. These findings shed new light on how vitamin D enhances re-epithelialization during wound repair by targeting EpSCs.

Vitamin D receptor (VDR) is an essential receptor in the VD3 signaling pathway and wound healing (8). VD3 binds and activates the nuclear VDR, which regulates the transcription of target genes (23,24). Mechanistically, we propose that vitamin D promoted wound healing through PI3K signaling pathways. PI3K signaling pathway in EpSCs was significantly phosphorylated in the presence of VD3. Inhibitors of the PI3K signaling pathway further indicated that PI3K is an important signaling pathway for the proliferation and differentiation of EpSCs.

While our findings highlighted the efficacy of vitamin D in wound repair, additional clinical work is needed to translate these results. Vitamin D deficiency is highly prevalent worldwide, estimated to affect over 1 billion people (25). Many elderly patients at high risk of chronic wounds are deficient in vitamin D (26). Our study provides a robust scientific rationale for correcting vitamin D insufficiency to support optimal wound healing, particularly in high-risk groups like the elderly and diabetics. However, clinical trials remain necessary to establish the safety and efficacy of vitamin D supplementation for wound therapy. Factors like optimal mode of delivery, dosage, timing, and patient stratification should be determined. With further validation, vitamin D therapy could provide a simple, inexpensive intervention to improve healing outcomes.

## Conclusions

Our study demonstrated that vitamin D is a potent promoter of cutaneous wound healing by stimulating EpSC proliferation and differentiation through PI3K activation. Our findings provided valuable insight into the therapeutic window of topical vitamin D for wound healing. Clinical doses should be carefully tailored to achieve maximal benefits without adverse effects.

## Supplementary Material

Click to view [pdf].
