# Supplementary material for: 1α,25-Dihydroxyvitamin D3 accelerates skin wound re-epithelialization by promoting epidermal stem cell proliferation and differentiation through PI3K activation: an in vitro and in vivo study
Source: Braz J Med Biol Res. 2025 Mar 3;58:e14121. doi: 10.1590/1414-431X2025e14121 (PMC11884782; doi:10.1590/1414-431X2025e14121)

Figure S1. The subcutaneous vitamin D injection sites in the murine full-thickness skin defect model.

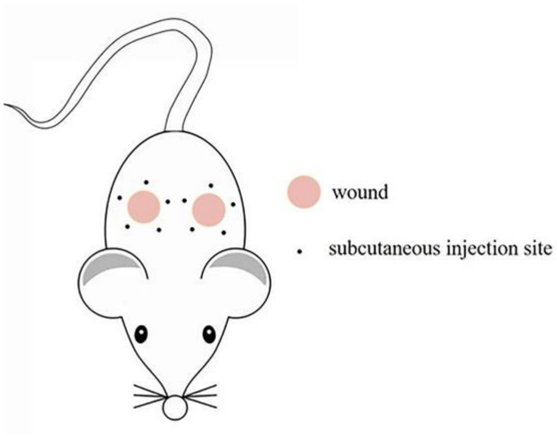

Figure S2. Pathological study of *in vivo* potential toxicity of the four doses of vitamin D on liver and kidney at 21 days post-wounding (scale bars: 100 μm).

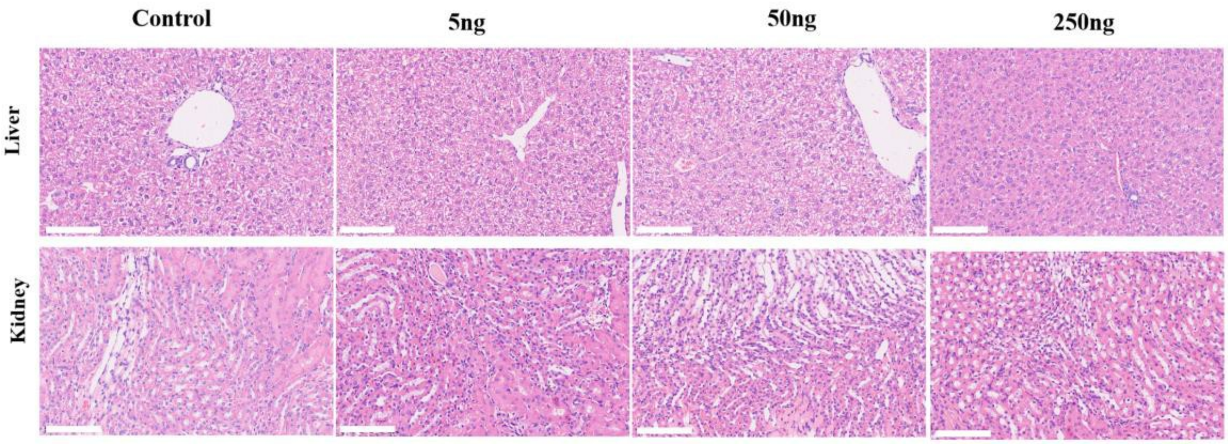

Supplement: Supplementary file 1 [file 1414-431X-bjmbr-58-e14121-suppl.pdf]
